# Molecular characteristics and risk factor analysis of *Staphylococcus aureus* colonization put insight into CC1 colonization in three nursing homes in Shanghai

**DOI:** 10.1371/journal.pone.0253858

**Published:** 2021-10-07

**Authors:** Wei-Ping He, Fei-Fei Gu, Ji Zhang, Xin-Xin Li, Shu-Zhen Xiao, Qian Zeng, Yu-Xing Ni, Li-Zhong Han

**Affiliations:** 1 Department of Laboratory Medicine, Ruijin Hospital, Shanghai Jiao Tong University School of Medicine, Shanghai, China; 2 Department of Clinical Microbiology, Ruijin Hospital, Shanghai Jiao Tong University School of Medicine, Shanghai, China; 3 Department of Clinical Laboratory, Shanghai People’s Hospital of Putuo District, Shanghai, China; Suez Canal University, EGYPT

## Abstract

Research indicates that *Staphylococcus aureus* colonization in the elderly with predisposing risks is associated with subsequent infection. However, the molecular epidemiology and risk factors for *S*. *aureus* colonization among residents and staff in nursing homes (NHs) in China remain unclear. A multicenter study was conducted in three NHs in Shanghai between September 2019 and October 2019. We explored the prevalence, molecular epidemiology, and risk factors for *S*. *aureus* colonization. All *S*. *aureus* isolates were characterized based on antimicrobial resistance, virulence genes, multilocus sequence typing (MLST), staphylococcus protein A (*spa*) typing, and staphylococcal cassette chromosome *mec* (SCC*mec*) typing. NH records were examined for potential risk factors for *S*. *aureus* colonization. *S*. *aureus* and methicillin-resistant *S*. *aureus* (MRSA) isolates were detected in 109 (100 residents and 9 staff, 19.8%, 109/551) and 28 (24 residents and 4 staff, 5.1%, 28/551) subjects among 496 residents and 55 staff screened, respectively. Compared to methicillin-susceptible *S*. *aureus* isolates, all 30 MRSA isolates had higher resistance rates to most antibiotics except minocycline, rifampicin, linezolid, vancomycin, and teicoplanin. Sequence type (ST) 1 (21.3%) was the most common sequence type, and t127 (20.5%) was the most common *spa* type among 122 *S*. *aureus* isolates. SCC*mec* type I (70%) was the dominant clone among all MRSA isolates. CC1 (26/122, 21.3%) was the predominant complex clone (CC), followed by CC398 (25/122, 20.5%), CC5 (20/122, 16.4%) and CC188 (18/122, 14.8%). Female sex (OR, 1.70; 95% CI, 1.04–2.79; *P* = 0.036) and invasive devices (OR, 2.19; 95% CI, 1.26–3.81; *P* = 0.006) were independently associated with *S*. *aureus* colonization.

## Introduction

*Staphylococcus aureus* is a gram-positive bacterium known to secrete infection-related toxins and invasive enzymes such as hemolysin, Panton-Valentine leukocidin, plasma-coagulase, and enterotoxins [1]. Infections caused by *S*. *aureus* range from skin and soft tissue infections (SSTIs) to invasive diseases, such as endocarditis, lung abscesses, and osteomyelitis, especially in hospitals and community settings [2–4]. In fact, *S*. *aureus* often colonizes asymptomatically on different body sites in healthy individuals, which significantly increases the chances of infection by providing a pathogen reservoir [5]. Methicillin-resistant *S*. *aureus* (MRSA) was first reported at a hospital in England in 1961, and it quickly became an important global pathogen [6]. MRSA is resistant to all currently available β-lactam antimicrobial agents, including β-lactamase-stable penicillins, and cephalosporins, which presents a challenge in terms of treatment and infection control [7]. To date, virulent MRSA strains are prevalent in the general community [8]. Furthermore, multidrug resistance has increased globally and is now considered a public health threat. Several previous studies revealed the emergence of multidrug-resistant bacterial pathogens from different origins especially birds, animals, and food chains which may be transmitted to human consumers resulting in severe illness [9–11]. Nursing homes (NHs) are implicated as important regional reservoirs of *S*. *aureus* [12], which poses a threat to elderly individuals [13]. Old and frail adults receiving NH care are at a higher risk for *S*. *aureus* infection due to age-related immune senescence, accumulation of comorbid conditions, impaired mobility, and frequent hospital admissions [14]. Living in small NH facilities was independently associated with MRSA colonization among residents, as reported in a Brazilian study [13]. Transmission dynamics within NHs may be explained by frequent close contact and potentially via fomite contamination in crowded living environments, shared physical therapy devices, shared bath equipment, and group dining facilities [15]. There are also frequent daily contacts between the staff and the residents. Gowns and gloves should be used during specific care activities, especially for residents with chronic skin issues such as pressure ulcers, which have a higher transmission risk [16]. Thus, it is necessary to evaluate the *S*. *aureus* colonization rate of NH staff as well.

Various *S*. *aureus* clones circulate in different countries or regions associated with specific geographical locations. CA-MRSA (community-associated MRSA) is characterized by strong pathogenicity, a broad drug resistance spectrum, a high drug resistance rate, and complex drug resistance mechanisms [17], which have been studied extensively. For example, the ST1 and ST8 clones are mainly found in the United States and Canada, while ST80 clones are more prevalent in Europe; moreover, ST59 clones are the most common MRSA clones in China and several other Asian countries [18]. Some STs are significantly associated with the occurrence of complications, disease severity, and mortality [19]. Therefore, adding improved surveillance for the molecular characteristics of *S*. *aureus* in NHs perhaps will better predict the prognosis of subsequent infections. Most epidemiological studies of *S*. *aureus* performed focus on MRSA emergence and spread in healthcare settings. However, few microbiological screening studies have been performed within NHs in China or regarding the molecular epidemiology and risk factors for *S*. *aureus* colonization. CC1 was found to be the predominant clone among residents in seven NHs in Shanghai in 2015 [20]. A further confirmatory investigation was conducted in 2019 in three NHs in Shanghai with staff being screened concurrently. The present study of *S*. *aureus* in NHs is important for resident health and for providing a better understanding of the successful epidemic clones in NHs, which play an important role in medical care [21].

The objective of this study was to explore the risk factors for *S*. *aureus* colonization among residents and to investigate the molecular epidemiology of *S*. *aureus* isolates derived from residents and staff in three NHs in Shanghai. The predictors identified will help form infection control strategies for minimizing *S*. *aureus* prevalence and transmission in NHs, thus reducing the social and economic impact of NH-associated *S*. *aureus* in nearby hospitals and the local community.

## Materials and methods

### Settings and participants

This cross-sectional study was performed between September 2019 and October 2019, in three NHs in Shanghai, China: one in the Jingan District, and the other two in the Putuo District. This study was a descriptive study that focused on the risk factors for *S aureus* and MRSA carriage and epidemiological surveillance of *S*. *aureus* colonization in specimens sourced from nasal, axillary, and skin samples from residents in three NHs in Shanghai. There are 35 and 43 registered NHs in Jingan District and Putuo District, respectively, that were issued business qualification certificates by the local district government. All three participating NHs (NH1, NH2, and NH3) are privately owned facilities containing 167, 320, and 180 beds, respectively. NHs were randomly selected and asked to participate in the study before it started in 2019. The inclusion criteria that were applied to participant: residents who signed the informed consent, had a normal body temperature during the survey phase, and had no obvious symptoms of infectious disease. Paper-based informed consent was signed by residents enrolled or by a statutory agent for residents with a cognitive deficit. Skin swabs from the wound surface were also collected if the residents were suffering from pressure sores or other skin lesions. Each of the 496 residents was surveyed with a real-name questionnaire to assess age, sex, comorbidities, any invasive devices being used, hospital admissions during the preceding 12 months, previous surgery, and antimicrobial usage in the previous 3 months. Data related to NH information included the number of resident beds, staffing ratio (staff per 10 beds), average monthly expenditure per resident, and sanitary conditions.

### Sample collection

This study was approved by the Ruijin Hospital Ethics Committee (Shanghai Jiao Tong University School of Medicine) and the approval code was RJ2019NO140-1. A total of 551 individuals (496 residents and 55 nursing staff), aged 40–102 years, in the three NHs were screened for *S*. *aureus*. All swabs were cultured on blood agar for 24 h at 37°C. Further analysis was based on morphology, Gram staining, catalase and coagulase testing, mannitol fermentation experiment, and *S*. *aureus* chromogenic medium at a clinical microbiology laboratory [22, 23]. Species confirmation was performed using matrix assisted laser desorption/ionization-time of flight (MALDI-TOF) mass spectrometry (VITEK^®^MS, bioMérieux, France) [24].

### Antimicrobial susceptibility testing

Antimicrobial susceptibility testing was performed on all confirmed isolates using the Kirby-Bauer disk-diffusion method [25] following the Clinical and Laboratory Standard Institute (CLSI) standards issued in 2019 [26]. In addition, referring to relevant research [20, 27, 28], the isolates were tested for susceptibility to the following antibiotics: penicillin (10 units), cefoxitin (30 μg), gentamicin (10 μg), kanamycin (30 μg), tobramycin (10 μg), erythromycin (15 μg), tetracycline (30 μg), teicoplanin (30 μg), minocycline (30 μg), ciprofloxacin (5 μg), clindamycin (2 μg), sulfamethoxazole-trimethoprim (25 μg), chloramphenicol (30 μg), rifampicin (5 μg), quinupristin-dalfopristin (15 μg), linezolid (30 μg), fusidic acid (10 μg), fosfomycin (200μg), and mupirocin (200 μg). The minimum inhibitory concentration (MIC) of vancomycin was determined using the agar dilution method [25]. *Staphylococcus aureus* ATCC25923 and ATCC29213 were used as quality controls for the disk diffusion tests and MIC detection, respectively. The identification of MRSA mainly depends upon the resistance to cefoxitin and the detection of the *mecA* gene.

### Detection of toxin genes

DNA was extracted using the simplified alkaline-lysis method [29]. Several clinically significant toxin genes [30, 31], including *sea-see* and *seg-sej* (which encode staphylococcus enterotoxin SEA-SEE and SEG-SEJ, respectively), *lukS/F-PV* (which encodes Panton-Valentine leucocidin), *tst* (which encodes toxic shock syndrome toxin-1), and *eta* and *etb* (which encode exfoliative toxin A and B, respectively), were analyzed by PCR assay. The PCR reactions consisted of 12.5 μL 2×Taq Master Mix (Accurate Biology Hunan, China), 1 μL forward primer (10 μM), 1 μL reverse primer (10 μM), 2 μL DNA template (200 ng/μL–600 ng/μL), and 8.5 μL ddH_2_O. The PCR conditions were determined and adjusted according to primers and amplicon lengths as previously described [32]. The reactions were run for 30 cycles (each cycle included 94°C for 30 s, 55°C for 30 s, and 72°C for 1 min), with initial hot start (94°C for 10 min) and final extension (72°C for 10 min). All the primers used in this study were synthesized by Sangon Biotech (Shanghai, China). We added 5 μL PCR products into each well, and the agarose gel electrophoresis ran for 40 min in 0.5×TAE solution under 110 v voltage. The PCR products were detected in a 1.5% agarose gel stained with ethidium bromide and visualized under UV light.

### Molecular typing

All *S*. *aureus* isolates were characterized by *staphylococcus* protein A (*spa*) typing [33] multilocus sequence typing (MLST) [34], and staphylococcal cassette chromosome *mec* (SCC*mec*) typing, as previously described [20]. The *spa* types and STs were assigned by uploading Sanger dideoxy DNA sequencing into online databases (http://spa.ridom.de/index.shtml; https://pubmlst.org/). A minimum spanning tree was generated by the PHYLOViZ web server based on the ST types (http://www.phyloviz.net). PCR assays were performed to identify the SCC*mec* structural types. The reagents used for PCR were the same as described above, so as the conditions for gel electrophoresis.

### Statistical analysis

The data were collected using Epidata 3.1 (Epidata Association, Odense, Denmark) and exported to SAS (version 8.2, SAS Institute Inc. Cary, NC) and SPSS (version 21, IBM-SPSS Inc. Armonk, NY) software for further analysis. Data regarding colonized and uncolonized NH residents were analyzed using univariate analysis with the χ^2^ test or Fisher exact test for categorical variables, and Student’s *t*test or the Mann-Whitney U test for continuous variables. Multilevel regression analysis was performed using SPSS, and multivariate logistic regression analysis was performed with the forward likelihood ratio logistic-regression model included in SAS to identify variables independently related to *S*. *aureus* and MRSA colonization. All tests performed were two-sided, and *P*<0.05 was considered statistically significant.

## Results

### Prevalence of *S*. *aureus* colonization and overview of the NHs

A total of 551 nasal swabs, 551 axillary swabs, and 40 skin swabs were collected from 551 individuals (496 elderly residents and 55 nursing staff) in three nursing homes in Shanghai. *S*. *aureus* colonization was detected in 100 residents and 9 nursing staff. The overall prevalence of *S*. *aureus* in all subjects was 19.8% (109/551). *S*. *aureus* isolates from 96 residents were detected in only one sample area (nasal, *n* = 77; axillary, *n* = 18; and skin, *n* = 1). Additionally, *S*. *aureus* isolates from 13 residents were detected in 2 of 3 sample areas (nasal and axillary, *n* = 12; axillary and skin, *n* = 1). No *S*. *aureus* isolates were detected in all three sample areas. A total of 88 *S*. *aureus* isolates were collected from 551 nasal swabs (88/551, 15.97%), including 22 MRSA isolates (22/551, 3.99%). Thirty-two *S*. *aureus* isolates were collected from 551 axillary swabs (32/551, 5.8%), including eight MRSA isolates (8/551, 1.45%). Two *S*. *aureus* isolates were collected from 40 skin swabs (2/40, 5%), and no MRSA was detected. A total of 109 (100 residents and 9 staff) and 28 (24 residents and 4 staff) subjects were found to be colonized by *S*. *aureus* and MRSA, respectively. Hence, the overall prevalence of *S*. *aureus* and MRSA colonization was 19.8% and 5.1% in the NHs.

In addition, 113 *S*. *aureus* isolates were collected from 100 of the 496 residents screened, including 26 MRSA isolates, and the overall prevalence of *S*. *aureus* and MRSA colonization among residents was 20.2% (100/496) and 4.8% (24/496), respectively. Two different genetic phenotypes of *S*. *aureus* isolates were obtained from three residents respectively, including one MRSA isolate. Moreover, nine *S*. *aureus* isolates were collected from 55 staff screened including four MRSA isolates, and the overall prevalence of *S*. *aureus* and MRSA among staff was 16.4% and 7.3%, respectively.

Among the 496 residents, 64.7% were women and the median age was 87 (range, 40–102; interquartile range [IQR], 79.0–90.5). The average monthly expenditure per resident ranged from ¥ 4550-4700(RMB), and the patient: staff ratio was 1.26–2.05 (Table 1). The median age of resident *S*. *aureus* and MRSA carriers was 86 (range, 64–99; IQR, 79.5–90) and 86 (range, 67–95; IQR, 75–89), respectively.

**Table 1:** General information of three nursing homes, including residents and staff, *Staphylococcus aureus* and MRSA carriers.

	Bed capacity (*n*)	Included residents (%)	*S*. *aureus* carriers (%)	MRSA carriers (%)	Staff (n)	Included staff (%)	*S*. *aureus* carriers (%)	MRSA carriers (%)	Average monthly expenditure per resident (¥)	Sanitary level	Staff per 10 beds
NH1	167	123 (73.7)	21 (17.1)	6 (4.9)	30	18 (60)	2 (11.1)	1 (5.6)	4550	3	2.05
NH2	320	245 (76.6)	40 (16.3)	8 (3.3)	37	20 (54.1)	3 (15)	2 (10)	4700	1	1.26
NH3	180	128 (71.1)	39 (30.5)	10 (7.8)	34	17 (50)	4 (23.5)	1 (5.9)	4700	2	2.14
Total	667	496 (74.4)	100 (20.2)	24 (4.8)	101	55 (54.5)	9 (16.4)	4 (7.3)			

Sanitary level:

Level 1: The room has an independent bathroom and can take a shower with a fixed bath time.

Level 2: There is an independent bathroom and shower in the room, but the bath time is not fixed.

Level 3: There is a separate bathroom in the room, but no shower and the bath time is not fixed.

### Antimicrobial resistance

Among the 113 *S*. *aureus* isolates collected from the elderly residents, 26 (23%) were MRSA and 87 (77%) were methicillin-susceptible *Staphylococcus aureus* (MSSA). Only one MRSA isolate was susceptible to penicillin among 26 MRSA. All resident isolates were susceptible to minocycline, sulfamethoxazole-trimethoprim, rifampicin, linezolid, vancomycin, and teicoplanin. Among the nine *S*. *aureus* isolates collected from the nursing staff, four strains (44%) were MRSA and five (56%) were MSSA. All *S*. *aureus* isolates from staff were susceptible to minocycline, chloramphenicol, rifampicin, quinupristin-dalfopristin, linezolid, fusidic acid, vancomycin, and teicoplanin. Compared to MSSA isolates, all 30 MRSA isolates had higher resistance rates to most antibiotics except minocycline, rifampicin, linezolid, vancomycin, and teicoplanin. The antibiotic resistance rates for all 122 *S*. *aureus* isolates (113 from residents and 9 from nursing staff) are presented in Table 2.

**Table 2:** The antimicrobial resistance rates of *Staphylococcus aureus* isolates obtained from residents and nursing staff in three nursing homes in Shanghai.

Antibiotic	Resistance rate					
	Residents			Nursing staff		
	Total (*n* = 113)	MSSA (*n* = 87)	MRSA (*n* = 26)	Total (*n* = 9)	MSSA (*n* = 5)	MRSA (*n* = 4)
Penicillin	89.4%	87.4%	96.2%	88.9%	80%	100%
Cefoxitin	23%	0	100%	44.4%	0	100%
Gentamicin	0.88%	0	3.8%	11.1%	0	25%
Kanamycin	9.7%	3.4%	30.8%	33.3%	0	75%
Tobramycin	6.2%	0	26.9%	33.3%	20%	50%
Erythromycin	39.8%	37.9%	46.2%	55.6%	40%	75%
Tetracycline	11.5%	10.3%	15.4%	22.2%	20%	25%
Sulfamethoxazole-trimethoprim	0	0	0	11.1%	0	25%
Chloramphenicol	2.7%	0	11.5%	0	0	0
Quinupristin-dalfopristin	1.8%	1.1%	3.8%	0	0	0
Fusidic acid	2.7%	1.1%	7.7%	0	0	0
Fosfomycin	11.5%	8%	23.1%	22.2%	0	50
Mupirocin	21.2%	20.7%	23.1%	44.4%	20%	75%
Ciprofloxacin	25.7%	17.2%	53.8%	44.4%	40%	50%
Clindamycin	3.5%	2.3%	7.7%	11.1%	0	25%
Minocycline	0	0	0	0	0	0
Rifampicin	0	0	0	0	0	0
Linezolid	0	0	0	0	0	0
Vancomycin	0	0	0	0	0	0
Teicoplanin	0	0	0	0	0	0

### Toxin genes

The toxin genes *eta*, *etb*, *see*, and *lukS/F-PV* have not been detected in any *S*. *aureus* isolate collected from residents. *sec* was detected most frequently among the screened toxin genes, occurring in 31 isolates (31/113, 27.4%). Forty-four isolates did not have any of the virulence genes including seven MRSA isolates, which were all SCC*mec* V. *sea* and *seh* were found more frequently among MRSA isolates than among MSSA isolates (*P* = 0.0011, *P* = 0.0313, respectively). The toxin genes *sed* and *sej* were detected only in MSSA isolates, but there was no statistical difference between MSSA isolates and MRSA isolates (*P*>0.05). Detailed results about toxin genes are listed in Table 3.

**Table 3:** Prevalence of toxin genes among *Staphylococcus aureus* isolates obtained from residents in three nursing homes in Shanghai.

Toxin genes	Positive rate (%)			*P* value
	Total (*n* = 113)	MSSA (*n* = 87)	MRSA (*n* = 26)	
*lukS/F-PV*	0	0	0	-
*tst*	12 (10.6)	11 (12.6)	1 (3.8)	0.2902
*sea*	27 (23.9)	14 (16.1)	13 (50)	0.0011
*seb*	13 (11.5)	7 (8)	6 (23.1)	0.072
*sec*	31 (27.4)	20 (23)	11 (42.3)	0.0779
*sed*	9 (8.0)	9 (10.3)	0	0.1150
*see*	0	0	0	-
*seg*	24 (21.2)	22 (25.3)	2 (7.7)	0.0601
*seh*	25 (22.1)	15 (17.2)	10 (38.5)	0.0313
*sei*	23 (20.4)	21 (24.1)	2 (7.7)	0.095
*sej*	9 (8.0)	9 (10.3)	0	0.1150
*eta*	0	0	0	-
*etb*	0	0	0	-

*lukS/F-PV*, gene encoding Panton-Valentine leukocidin

*tst*, gene encoding toxic shock syndrome toxin 1

*eta* and *etb*, gene encoding exfoliative toxin A and B

*sea*-*see* and *seg*-*sej*, gene encoding staphylococcal enterotoxins SEA-SEE and SEG-SEJ

*P* value, two-sided *P* value calculated by the chi-square or Fisher’s exact test as appropriate.

### Molecular typing

**Fig 1:**
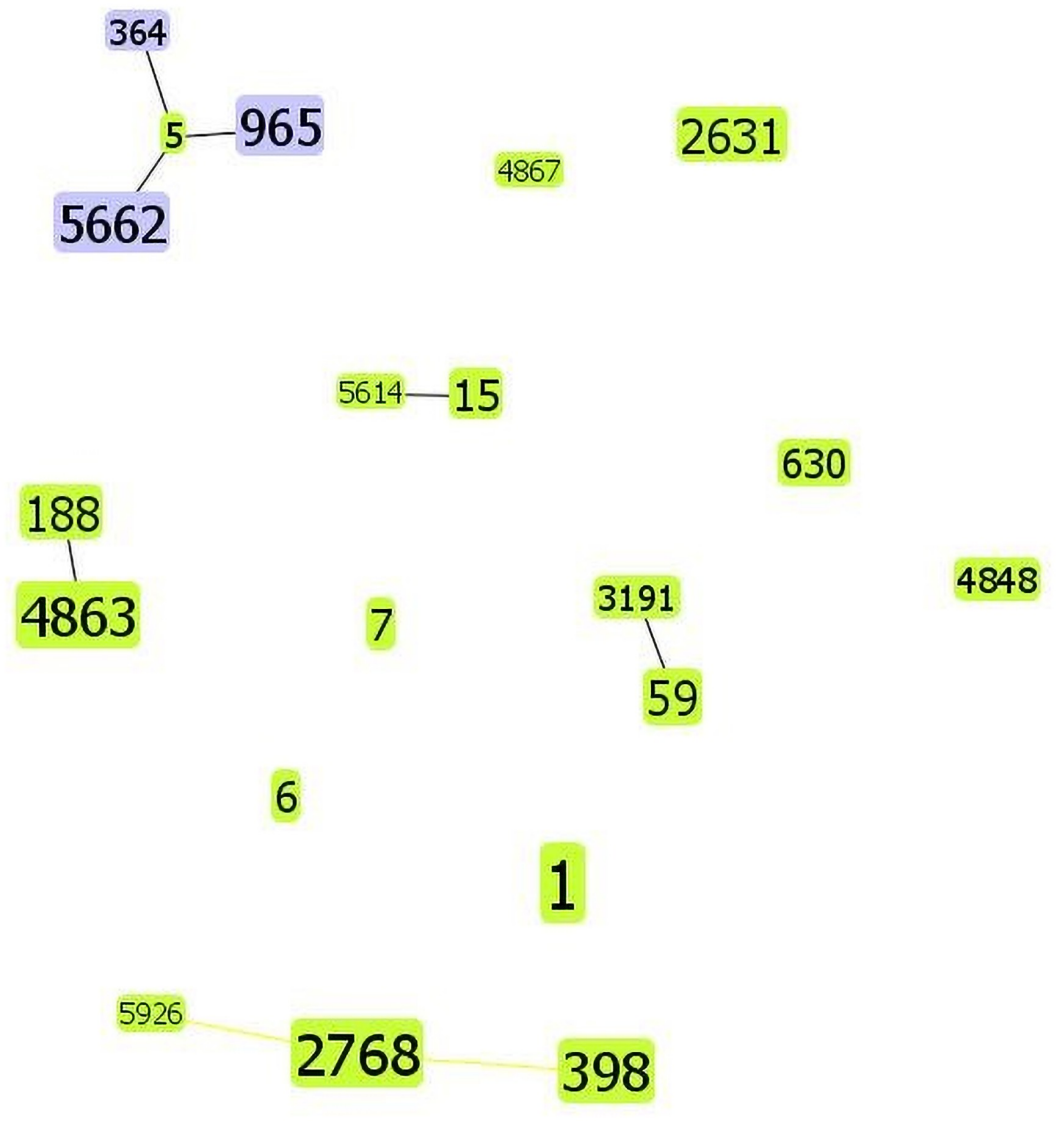
The diagram produced by PHYLOViZ with the stringent (default) group definition based on the MLST data of this study, representing the relationships of 122 *S*. *aureus* isolates.

In total, 20 sequence types (STs) were detected in this study. ST1 (26/122, 21.3%) was the most common ST, followed by ST2768 (14/122, 11.5%), ST4863 (12/122, 9.8%) and ST398 (10/122, 8.2%). Moreover, ST1 was the most common ST in both nasal carriers (15/88, 17.1%) and axillary carriers (10/32, 31.3%). Only two *S*. *aureus* isolates were obtained from the skin carriers: ST1 and ST2768. Five MSSA isolates could not be *spa* typed. Two of these belonged to ST188, and the remaining three isolates belonged to ST15, ST4848, and ST4863, respectively. Thirty-two *spa* types were identified in all 122 *S*. *aureus* isolates. t127 (25/122, 20.5%) was the most common *spa* type, followed by t571 (14/122, 11.5%) and t189 (11/122, 9.0%). t127 was the most common *spa* type in both nasal carriers (15/88, 17.0%) and axillary carriers (9/32, 28.1%). In addition, 21 SCC*mec* I (21/30, 70%), one SCC*mec* II (1/30, 3.3%), and eight SCC*mec* V (8/30, 26.7%) type MRSA isolates were identified (Tables 4 and 5). When STs and *spa* typing analysis were combined, the predominant clones were ST1-t127 (22/113, 19.5%), followed by ST2768-t571 (14/113, 12.4%) and ST4863-t189 (11/113, 9.7%) among residents. In addition, ST1-t127 (3/9,33.3%) was the most prevalent clone among staff. Among MRSA isolates, SCC*mec* I-ST1-t127 was the most common clone, accounting for 38.5% (10/26, residents) and 50% (2/4, staff). As shown in Fig 1, the diagram produced by PHYLOViZ using a stringent (default) group definition, each number represents an ST type, in the 122 *S*. *aureus* isolates. CC1 (26/122, 21.3%) was the predominant complex clone (CC), followed by CC398 (25/122, 20.5%), CC5 (20/122, 16.4%) and CC188 (18/122, 14.8%).

**Table 4:** Molecular characteristics of *Staphylococcus aureus* isolates obtained from residents living in three nursing homes in Shanghai.

ST	Isolates (*n*)	MSSA, (*n*)	MRSA SCC*mec* Type (*n*)	*spa* type (*n*)	Virulence factors (*n*)
1	23	13	I (10)	t127(10)	*sea*(7), *sec* (10), *seh*(10)
				t127(12), t948(1)	*sea*(10), *sec*(7), *seh*(13)
15	4	4		t11579(2), t279(1), NT (1)	None
188	6	6		t13446(1), t2186(2), t2883(1), t4445(1), NT(1)	*tst*(2), *seb*(1), *sec*(2)
2631	5	5		t164(5)	*seb*(1), *seg*(5), *seh*(1), *sei*(4)
2768	14	14		t571(14)	None
3191	2	1	I (1)	t437(1)	*sea*(1), *seb*(1)
				t437(1)	*seb*(1)
364	2	0	I (2)	t002(2)	*tst*(1), *seb*(1), *sec*(1), *seg*(2), *sei*(2)
398	9	4	V (5)	t011(1), t034(4)	None
				t034(2), t1170(1), t1184(1)	None
4848	2	2		NT(2)	*sea*(1), *seb*(1)
4863	12	11	II (1)	t189(1)	*seb*(1)
				t189(10), NT(1)	*tst*(2), *seb*(3), *sec*(2), *seh*(1)
5	2	2		t548(1), t6662(1)	*sec*(1), *sed*(1), *seg*(2), *sei*(2), *sej*(1)
5614	1	1		t084(1)	None
5662	8	8		t442(8)	*sed*(8), *seg*(8), *sei*(8), *sej*(8)
59	5	0	I (5)	t163(3), t172(2)	*sea*(5), *seb*(3)
5926	1	1		t4389(1)	None
6	4	4		t2360(1), t701(3)	*sea*(3), *sec*(1)
630	3	2	V (1)	t4549(1)	None
				t377(2)	None
7	3	2	V (1)	t796(1)	None
				t1685(2)	None
965	7	7		t062(7)	*tst*(7), *sec*(7), *seg*(7), *sei*(7)

ST, sequence type by multi-locus sequence typing; SCC*mec*, Staphylococcal cassette chromosome *mec*; *spa*, Staphylococcus protein A gene; NT, not-typeable; None, no virulence gene detected.

**Table 5:** Molecular characteristics of *Staphylococcus aureus* isolates obtained from nursing staff caring for residents in three nursing homes in Shanghai.

ST	Isolates (*n*)	MSSA (*n*)	MRSA SCC*mec* Type (*n*)	*spa* type (*n*)	Virulence factors (*n*)
1	3	1	I (2)	t127(2)	*seg*(1), *seh*(1), *sei*(1)
				t127(1)	*sea*(1), *sec*(1), *seh*(1)
2631	1	1		t164(1)	*seg*(1), *sei*(1)
398	1	1		t1184(1)	None
4867	1	1		t078(1)	*seb*(1), *seg*(1), *sei*(1)
59	1	0	I (1)	t163(1)	*sea*(1), *sec*(1)
7	1	0	V (1)	t796(1)	None
965	1	1		t062(1)	*tst*(1), *sec*(1)

ST, sequence type by multi-locus sequence typing; SCC*mec*, Staphylococcal cassette chromosome *mec*; *spa*, Staphylococcus protein A gene; None, no virulence gene detected.

Three residents were each colonized by two distinct genotype clones, i.e., different *S*. *aureus* clones were collected from two different sample areas. The genetic phenotype distribution for these three *S*. *aureus* colonization residents is shown in Table 6.

**Table 6:** Molecular characteristics of six *Staphylococcus aureus* strains collected from three residents.

Resident	Sample type	*spa* type	ST	SCC*mec* type	virulence factors	NH
405	nasal swabs	t279	ST15		None	3
	axillary swabs	t084	ST5614		None	3
205	nasal swabs	t164	ST2631		*seb*(1), *seg*(1), *seh*(1), *sei*(1)	2
	axillary swabs	t442	ST5662		*sed*(1), *seg*(1), *sei*(1), *sej*(1)	2
369	nasal swabs	t034	ST398	V	None	3
	axillary swabs	t571	ST2768		None	3

ST, sequence type by multi-locus sequence typing; SCC*mec*, Staphylococcal cassette chromosome *mec*; *spa*, Staphylococcus protein A gene; None, no virulence gene detected; NH, nursing home.

### Risk factor analysis

In the univariate analysis, the significant risk factors for MRSA colonization were previous hospitalization (odds ratio [OR], 2.50; 95% confidence interval [CI], 1.07–5.82; *P* = 0.029), invasive devices (OR, 3.45; 95% CI, 1.45–8.18; *P* = 0.003), senile pruritus (OR, 2.72; 95% CI, 1.09–6.83; *P* = 0.027), renal insufficiency (OR, 4.91; 95% CI, 1.69–14.28; *P* = 0.001), and diabetes mellitus (OR, 3.72; 95%CI, 1.59–8.70; *P* = 0.001). The only significant risk factor for *S*. *aureus* colonization was invasive devices (OR, 1.96; 95% CI, 1.14–3.36; *P* = 0.014). The results of univariate statistical analysis are shown in Table 7. In terms of NH characteristics, sanitation levels and the number of staff were both significant factors for *S*. *aureus*, but not MRSA. The definition of the sanitation level is shown in Table 1. The average monthly expenditure per resident was not a significant factor for *S*. *aureus* or MRSA colonization.

**Table 7:** Analysis of univariate risk factors for *Staphylococcus aureus* and MRSA carriers in nursing homes.

	Total	*Staphylococcus aureus* carriers			MRSA carriers		
	n	n (%)	OR (95% CI)	*P* value	n (%)	OR (95% CI)	*P* value
Demographics							
Age, years, median (IQR)	87 (79.0–90.5)	86 (79.5–90.0)	-	0.0980	86 (75.0–89.0)	-	0.4040
gender							
male	175	28 (16.0%)	0.658 (0.407–1.066)	0.0884	9 (5.1%)	1.106 (0.474–2.582)	0.8159
female	321	72 (22.4%)	1.518 (0.938–2.458)	0.0884	15 (4.7%)	0.904 (0.387–2.111)	0.8159
Comorbidities							
Cardio-cerebrovascular disease	449	93 (20.7%)	1.493 (0.648–3.440)	0.3446	22 (4.9%)	1.159 (0.264–5.091)	1
Digestive system disease	64	15 (23.4%)	1.250 (0.669–2.335)	0.4844	2 (3.1%)	0.601 (0.138–2.620)	0.7095
Respiratory disease	107	15 (14.0%)	0.583 (0.321–1.059)	0.0740	3 (2.8%)	0.506 (0.148–1.728)	0.3935
Respiratory infection	11	4 (36.4%)	2.316 (0.664–8.071)	0.3298	1 (9.1%)	2.009 (0.247–16.368)	1
Renal insufficiency	29	8 (27.6%)	1.553 (0.667–3.617)	0.3049	5 (17.2%)	4.912 (1.690–14.282)	0.0014
Urinary tract infection	9	1 (11.1%)	0.490 (0.061–3.963)	0.7920	1 (11.1%)	2.522 (0.303–21.022)	0.9194
Cancer	24	5 (20.8%)	1.044 (0.380–2.869)	0.9330	1 (4.2%)	0.849 (0.110–6.564)	1
Alzheimer’s disease	117	21 (17.9%)	0.831 (0.487–1.416)	0.4954	3 (2.6%)	0.449 (0.131–1.532)	0.2868
Diabetes	161	38 (23.6%)	1.360 (0.862–2.147)	0.1859	15 (9.3%)	3.722 (1.592–8.699)	0.0013
Impaired mobility	129	30 (23.3%)	1.286 (0.792–2.087)	0.3090	9 (7.0%)	1.760 (0.751–4.126)	0.1888
Senile pruritus	69	19 (27.5%)	1.623 (0.908–2.902)	0.1002	7 (10.1%)	2.723 (1.085–6.832)	0.0270
Antibiotic treatment	128	22 (17.2%)	0.912 (0.551–1.510)	0.7204	9 (7.0%)	1.780 (0.759–4.173)	0.1800
Medical history							
Prior hospitalization	204	47 (23.0%)	1.350 (0.868–2.099)	0.1822	15 (7.4%)	2.496 (1.070–5.819)	0.0293
Operation history	49	14 (28.6%)	1.679 (0.865–3.258)	0.1226	5 (10.2%)	2.500 (0.911–7.191)	0.0655
Invasive device	79	24 (30.4%)	1.958 (1.141–3.360)	0.0136	9 (11.4%)	3.446 (1.452–8.180)	0.0031
Nursing homes							
AMEPR, RMB, median (IQR)	4700 (4700–4700)	4700 (4550–4700)	-	0.3250	4700 (4625–4700)	-	0.9810
Staff per 10 beds	2.05 (1.26–2.14)	2.05 (1.26–2.14)	-	0.0040	2.05 (1.26–2.14)	-	0.0600
Sanitary level				0.0033			0.1514
1	245	40 (16.3%)	0.621 (0.398–0.970)	0.0356	8(3.3%)	0.496 (0.208–1.181)	0.1070
2	128	39 (30.5%)	2.205 (1.384–3.515)	0.0007	10 (7.8%)	2.143 (0.927–4.953)	0.0690
3	123	21 (17.1%)	0.766 (0.766–1.303)	0.3254	6 (4.9%)	1.011 (0.392–2.608)	0.9813

In multilevel logistic regression analysis, the result of the empty model (model *S*. *aureus* and model MRSA) showed that the intraclass correlation coefficient was 0.169 (*P* = 0.414) and 0.110 (*P* = 0.634), respectively. Multivariate logistic regression analysis was performed to identify any significant independent risk factors. The multivariate logistic regression analysis (Table 8) showed that the significant independent risk factors for MRSA colonization were invasive devices (OR, 2.70; 95% CI, 1.09–6.68; *P* = 0.032), senile pruritus (OR, 2.88; 95% CI, 1.09–7.59; *P* = 0.033), renal insufficiency (OR, 4.12; 95% CI, 1.31–12.95; *P* = 0.016), and diabetes (OR, 2.99; 95% CI, 1.24–7.20; *P* = 0.015). The equation of the final model was as follows: logit*P* = -4.1072 + 0.9909 device + 1.0559 senile pruritus + 1.4146 renal insufficiency + 1.0950 diabetes mellitus, and the -2log likelihood was 192.189 (*P* = 0.0001). The significant independent risk factors for *S*. *aureus* colonization were female sex (OR, 1.70; 95% CI, 1.04–2.79; *P* = 0.036) and invasive devices (OR, 2.19; 95% CI, 1.26–3.81; *P* = 0.006). The equation of the final model was: logit*P* = -1.8798 + 0.5297 female sex + 0.7820 invasive device, and the -2log likelihood was 498.609 (*P* = 0.006).

**Table 8:** Multivariate logistic regression analysis of risk factors associated with *Staphylococcus aureus* and MRSA colonization among residents in three nursing homes.

Risk factor	*S*. *aureus* carriers			MRSA carriers		
	B*	OR (95% CI)	*P* value	B*	OR (95% CI)	*P* value
Female sex	0.5297	1.698 (1.035–2.787)	0.0361	-	-	-
Invasive device	0.7820	2.186 (1.256–3.805)	0.0057	0.9909	2.694(1.087–6.676)	0.0324
Senile pruritus	-	-	-	1.0559	2.875(1.088–7.594)	0.0331
Renal insufficiency	-	-	-	1.4146	4.115(1.308–12.945)	0.0156
diabetes	-	-	-	1.0950	2.989(1.241–7.199)	0.0146
constant	-1.8798	-	<0.0001	-4.1072	-	< .0001

* Partial regression coefficient.

## Discussion

About a third of healthy individuals in the community are colonized by *S*. *aureus* in the nostrils [2]. Nasal carriage of *S*. *aureus* has been associated with subsequent infection [35], and carriers are an important source of infection spread in communities. Infections caused by MRSA tend to be either widespread or are local outbreaks, which are difficult to treat, have high fatality rates, and increase the socio-economic and medical burden [6]. Compared with healthcare-associated MRSA (HA-MRSA) infections, CA-MRSA infections can occur in healthy individuals with no identified predisposing risk, suggesting that these strains may have greater virulence [36]. Nursing homes increase MRSA transmission and infection risk as a result of admission of elderly residents with premorbid conditions and weakened immune systems [37–39]. The MRSA colonization rate among residents in this study (24/496, 4.8%) was much lower than that in other studies showing 9.0% in Belgium [40], 9.04% in Saudi Arabia [41], and 14.5% in Crete; in Greece [12], the colonization rate we observed was slightly higher than that in Brazil (3.7%) [13] which may differ by geographic location.

Twenty STs and thirty-two *spa* types were found in 122 *S*. *aureus* isolates collected from the three NHs in Shanghai, indicating high genetic diversity and complexity. In our study, the most prevalent *S*. *aureus* lineages were CC1 (21.3%), CC398 (20.5%), CC5 (16.4%), and CC188 (14.8%) which was generally consistent with a 2014 study of seven NHs in Shanghai [20]. We then might be able to hypothesize that CC1 was the dominant clone during the past 5 years in NHs in Shanghai, which may point out the direction for our future research. However, CC1-SCC*mec* I MRSA is gradually replacing CC1-SCC*mec* IV-MRSA as the predominant MRSA clone. These results indicated that SCC*mec* I-MRSA, which was generally associated with HA-MRSA infection, might spread to NHs. Previous studies reported that HA-MRSA infection is generally accompanied with multidrug-resistance [42]. It is necessary to strengthen MRSA admission screening and take appropriate quarantine measures to prevent the spread of HA-MRSA in communities. Moreover, there was a consistent dominant clone in both residents and staff which may indicate the *S*. *aureus* transmission dynamics in NHs, was similar to previous study results. In addition, gloves and gown use is highly recommended [16]. CC1-MSSA is among the most frequently observed clonal lineage in animals in some African countries [43]. CC398, which has been identified as a colonizer or infectious agent in livestock, was the second most common CC in our study. The genetic background association between CC1 and CC398 needs further research. There was a strong association between ST 1 and *spa* type t127 in our present study and our 2014 study [20]. In addition, ST2768 was the second most common ST (14/113, 12.4%), and all ST2768 isolates were MSSA. Importantly, all 12 ST2768 isolates were collected from the residents in NH2, which indicates that NHs should strengthen the awareness of the residents and healthcare workers. ST2768 was first documented in Spain from an ulcer swab of a patient with a diabetic foot infection in 2016, as shown in the *S*. *aureus* MLST database (https://pubmlst.org/saureus/). ST188 and ST4863 belong to CC188, of which 66.7% (12/18) of isolates were ST4863. These isolates were all obtained from the residents in NH3. Notably, 11 ST4863 isolates were genotyped as (*spa*) t189, and the other one was untyped. Moreover, ST4863 was recently added to the PubMLST database by Jie Hong in 2018. This strain was identified in Jiangsu Province, which is west of Shanghai. This result suggests that we should further monitor the molecular characteristics of the *S*. *aureus* and work to find the relationship between genetic relatedness and neighborhood of emerging ST clones.

Among the 16 residents with MRSA typed SCC*mec* I, 10 residents had a history of recent hospitalization, which confirmed that recent hospitalization might be an important risk factor for MRSA colonization [13]. Among the four MRSA isolates collected from the staff, three MRSA isolates were identified as SCC*mec* I. This result was expected as Albrecht et al. [16] reported that MRSA transmission between residents and nursing staff often occurs during activities such as tidying beds, diaper changes, dressing changes, and transfers of position. In addition, nursing staff clothing and hands become contaminated with MRSA after caring for colonized residents. Thus, transmission was likely caused by direct contact with the contaminated hands of the nursing staff or the *S*. *aureus* isolates in the nursing staff survived in the environment and were transmitted after contact with the environment.

One hundred and thirteen *S*. *aureus* isolates were observed in 100 residents. Thirteen of 100 residents were colonized by *S*. *aureus* at two different sites. The molecular characteristics of the *S*. *aureus* isolates from different sites among the three resident groups were genetically different, as shown in Table 5. One MSSA isolate and one MRSA isolate were simultaneously obtained from one resident, and the MRSA strain was type SCC*mec* V, which indicates CA-MRSA [6]. Though it was suggested by Petry et al. [44] that in cases of wound or tissue samples with pending culture results, a negative MRSA nasal swab may influence the decision to withhold or discontinue MRSA-active screening. Samples from different sites should be collected when conducting *S*. *aureus* screening and evaluating the elimination of colonization [20, 45].

Several studies revealed that *lukS/F-PV* is more closely associated with skin and soft tissue infections [46, 47]. In this study, all *S*. *aureus* isolates were negative for genes that encode for Panton-Valentine leucocidin, enterotoxin E, and exfoliative toxins A and B. Interestingly, the frequency of *tst* in CC5 (9/13, 69.2%) isolates was significantly higher than that in other clones; only one isolate was MRSA, which may indicate that different *S*. *aureus* lineages have specific virulence gene patterns.

Similar to previous studies, invasive device presence was independently associated with *S*. *aureus* and MRSA colonization [12, 48, 49]. Although Stensen et al. reported that men have higher *S*. *aureus* nasal carrier rates than women [50], female sex was an independent risk factor for *S*. *aureus* colonization in the present study. In our previous study, we observed that the incidence of MRSA colonization was high in elderly patients with senile pruritus [51] which was confirmed in this study here. The link between renal insufficiency and MRSA colonization was also reported among hemodialysis patients [52]. Residents needing dialysis periodically go to the hospital, which may partially explain the increased risk. Notably, diabetic patients with specific pathological changes tend to have multiple complications. Previous studies suggest that the levels of MRSA colonization among diabetics with comorbidities in NHs or the wider community (patients and outpatients) reflects the extent of interaction with the healthcare system [53, 54].

The main limitation of our study is the inclusion of a relatively small number of subjects. Larger sample sizes from community NHs are required to accurately depict the prevalence of *S*. *aureus* colonization in Shanghai. Additionally, a limited number of staff members caring for residents in NHs was included in this study. The genetic relationship between isolates colonized among residents and staff needs further research. Finally, the colonization mechanism of CC1 *S*. *aureus* isolates among residents in NHs remains unclear. A future study using whole-genome sequencing may help determine the mechanisms underlying *S*. *aureus* colonization.

## Conclusions

The results provided by our study indicated that there are still measures we could take to control the high prevalence of *S*. *aureus* colonization in NHs. Strengthening the supervision and implementation of personal hygiene for resident individuals who may need assistance from nursing staff and staff individuals could reduce the source of infection. Furthermore, specific nursing management strategies for residents with predisposing factors for *S*. *aureus* colonization are indispensable such as rational drug use and regular screening.

## Supporting information

S1 Table Information and molecular characteristics of *S*. *aureus* isolates among residents and staff of three nursing homes in Shanghai. (XLSX)
